# Blood Metabolomics Analysis Identifies Differential Serum Metabolites in Elite and Sub-elite Swimmers

**DOI:** 10.3389/fphys.2022.858869

**Published:** 2022-05-05

**Authors:** Ming Cai, Chao Wu, Chen Jing, Xunzhang Shen, Mian He, Liyan Wang, Qi Guo, Yan Yan, Xu Yan, Ruoyu Yang

**Affiliations:** ^1^ Shanghai University of Medicine and Health Sciences Affiliated Zhoupu Hospital, Shanghai, China; ^2^ College of Rehabilitation Sciences, Shanghai University of Medicine and Health Sciences, Shanghai, China; ^3^ Foundation of Shanghai Vocational College of Agriculture and Forestry, Shanghai, China; ^4^ Shanghai Research Institute of Sports Science (Shanghai Anti-Doping Center), Shanghai, China; ^5^ School of Life Science, Qufu Normal University, Qufu, China; ^6^ Institute for Health and Sport (iHeS), Victoria University, Melbourne, VIC, Australia; ^7^ Australian Institute for Musculoskeletal Science (AIMSS), Melbourne, VIC, Australia; ^8^ Department of Medicine - Western Health, The University of Melbourne, Melbourne, VIC, Australia

**Keywords:** athletic status, metabolomics, metabolites, swimmers, nuclear magnetic resonance

## Abstract

**Objective:** Metabolites in body fluids, such as lactate, glucose, and creatinine, have been measured by conventional methods to evaluate physical function and performance or athletic status. The objectives of the current study were to explore the novel metabolite biomarkers in professional swimmers with different competition levels using nuclear magnetic resonance (NMR) metabolomics, and try to establish a model to identify the athletic status or predict the competitive potential.

**Methods:** Serum samples were collected from 103 elite and 84 sub-elite level Chinese professional swimmers, and were profiled by NMR analysis.

**Results:** Out of the thirty-six serum metabolites profiled, ten were associated with the athletic status of swimmers (with *p* < 0.05). When compared with sub-elite swimmers, elite swimmers had higher levels of high-density lipoprotein (HDL), unsaturated fatty acid, lactic acid, and methanol. Elite swimmers had lower levels of isoleucine, 3-hydroxybutyric acid, acetoacetate, glutamine, glycine, and α-glucose. A model with four metabolites, including HDL, glutamine, methanol, and α-glucose, was established to predict athletic status by adjusting with different covariates. The area under the curve (AUC) of the best model was 0.904 (95% CI: 0.862-0.947), with a sensitivity and specificity of 75.5 and 90.2%, respectively.

**Conclusion:** We have identified ten metabolite biomarkers with differentially expressed levels between elite and sub-elite swimmers, the differences could result from genetic or sports level between the two cohorts. A model with four metabolites has successfully differentiated professional swimmers with different competitive levels.

## Introduction

For competitive sports, identification of competition status or predicting the development trend of competition level has been a topic discussed in the fields of sports training, monitoring, and talent identification (Johnston et al., 2018). In some studies, certain metabolites in blood and urine, such as lactate, glucose, and creatinine have been measured by conventional methods to evaluate physical function and performance or athletic status (Papassotiriou and Nifli, 2018). Other metabolites have not been detected due to their low concentration in the blood or urine, which could still be important in the biological reactions of exercise processes (Alosco et al., 2020). More sensitive methods are in need to detect those lowly expressed metabolites, so that their potential important information will not be omitted.

Metabolomics is an important part of systems biology, apart from genomics, transcriptomics, and proteomics (Nicholson and Lindon, 2008; Baharum and Azizan, 2018). Nuclear magnetic resonance (NMR) is a commonly used analytical method in metabolomics (Markley et al., 2017; Ragavan and Merritt, 2019). The NMR method has a few advantages, such as simple sample pretreatment and the capacity to analyze several biological fluids including blood, urine, and saliva (Emwas, 2015; Bingol, 2018). Because of these advantages, NMR has been widely used in sports and exercise science (Sun et al., 2017; Heaney et al., 2019; Pitti et al., 2019).

Metabolomics analyses have been employed to monitor the metabolic profile of elite athletes. A pilot metabolomics analysis compared the metabolic profile between high- and moderate-endurance and power elite athletes, and reported that high-endurance and high-power athletes present a different metabolic profile, which includes metabolites related to energy production, fatty acid metabolism, oxidative stress, and steroid biosynthesis (Al-Khelaifi et al., 2018). Another study investigated the metabolic fluctuations in saliva samples of professional basketball players during a game, and showed that quarters 1 and 3 had similar saliva metabolic profiles, while quarters 2 and 4 also demonstrated similar saliva metabolic profiles, but metabolic profiles after quarters 1 and 3 were different from those after quarters 2 and 4 (Khoramipour et al., 2021). The metabolic data also suggested that the first and third quarters relied more on anaerobic energy contribution, whereas the second and fourth quarters utilize more aerobic energy (Khoramipour et al., 2021). These studies suggested that metabolic files can be altered after both acute exercise and chronic training. Therefore, metabolomics analyses can be utilized to identify athletic ability, training level, and state of a certain event athlete.

Swimming is a sporting event requiring great physical fitness (Burkett et al., 2018). The growth cycle of elite swimmers is very long; therefore, even small differences at a certain stage of athletes could affect their athletic capacity, which might result in them to be either elite athletes or sub-elite athletes (Haugen et al., 2019). Metabolites in body fluids have been used to monitor the training effects in swimmers. A study measured the urine metabolites before and after a swimming training session of elite swimmers with metabolomics analyses and reported peaks of ketone bodies, creatine, phosphate, and nitrogenous compounds after a 150 min training session (Khoramipour et al., 2021). Moreover, the metabolites of elite swimmers prior to the training session were different from those of controls (Khoramipour et al., 2021). The authors suggested the peaks of metabolites in urine can be used to evaluate and to adjust the physical training of elite swimmers (Moreira et al., 2018). However, it is still not clear whether there is any difference in the blood metabolomics characteristics between swimmers from different athletic statuses or levels. And if present, whether we can establish a prediction model for athletic status or level based on the different metabolites.

In this current study, we recruited swimmers from different athletic statuses as the research participants and investigated the characteristics of the blood metabolomics with the NMR method. With this cross-sectional study, we performed an untargeted metabolic analysis to determine the serum metabolites associated with athletic status in swimmers. Furthermore, we aimed to explore specific metabolites that could serve as biomarkers to identify the athletic status and evaluate athletes’ potential to achieve an elite level. By establishing the model with serum metabolites, coaches and researchers might be able to better assess the athletic status and competitive level of swimmers.

## Materials and Methods

### Ethics Approval

This study was conducted according to the Declaration of Helsinki and approved by the Ethics Committee at the School of Life Sciences, Fudan University, China. Written informed consent was obtained from all participants.

### Study Design and Participants

All participants (swimmers) were in their post competition recovery period. Two weeks before the blood sample collection, all swimmers adopted a training program with similar exercise volume and intensity (30 min of land exercises before swimming, including 15 min of stretching exercises and 15 min of relaxation exercises; swimming session lasting about 80–90 min, 4000 m swimming at around 60% of the maximum intensity; 15 min of relaxation exercises after the swimming session). The daily diet was carried out according to the unified recipe (a unified diet menu for athletes from Monday to Sunday at the training base), supervised by the coach in charge. In two weeks, athletes who took medicine did not follow the training program or who did not follow the diet were excluded from the study. After two weeks, all qualified swimmers (n = 187) were categorized into two groups (elite group and sub-elite group) according to their officially certified level of sports competition.

A total of 103 international- and national-level swimmers were from the Shanghai and Zhejiang professional swimming teams as the elite group. Athletes in the elite group have participated in international or national swimming competitions. There were 53 male athletes (height = 184.7 ± 5.2 cm, body mass = 78.7 ± 9.3 kg, age ranges: 18–29 years, training years: more than 10 years) and 50 female athletes (height = 171.8 ± 5.0 cm, body mass = 62.2 ± 6.3 kg, age ranges: 16–27 years, training years: more than 8 years).

Eighty-four first- and second-grade swimmers were from the Shanghai professional swimming team, Shanghai University of Sport, Shanghai Jiao Tong University, and Tong Ji University as the sub-elite group. Athletes in the sub-elite group have participated in provisional or universities swimming competitions. There were 52 male athletes (height = 180.1 ± 6.3 cm, body mass = 77.1 ± 10.1 kg, age ranges: 17–23 years, training years: more than 9 years) and 32 female athletes (height = 168.7 ± 4.9 cm, body mass = 59.8 ± 8.6 kg, age ranges: 16–22 years, training years: more than 8 years).

### Blood Samples Collection and Metabolomics Analysis

Blood samples (5 ml) were collected in tubes without anticoagulant from swimmers in the morning after overnight fasting. The samples were kept at room temperature for 30 min and then centrifuged at 4°C at 4000 rpm for 15 min (Centrifuge 5702R, Eppendorf AG, Hamburg, Germany). The serum samples were aliquoted into freezing tubes (Corning 430,659, 2 ml, United States), frozen immediately in liquid nitrogen, and stored at -80°C for around 1 week without a second freeze-thaw cycle before testing. On the day of NMR analysis, 170 ul serum sample was mixed with 340 μl PBS (phosphate buffer saline) in a 5 mm NMR tube and used directly for ^1^H NMR detection.

All NMR spectra were acquired at 298 K *via* Bruker AVIII 600 MHz NMR spectrometer (600.13 MHz for proton frequency), equipped with a cryogenic probe (Bruker Biospin, Germany). For the analysis of serum samples, we used the Carr-Purcell-Meiboom-Gill (CPMG) pulse sequence (RD-90°-(τ-180°-τ)n-ACQ), where τ = 350 ms and n = 100. A total of 32 transients for all samples were collected into 32K data points over a spectral width of 20 ppm with a 90° pulse length adjusted to 11.3 ms.

The free induction decays were multiplied by an exponential window function with the line-broadening factor of 1 Hz prior to Fourier transformation. Each spectrum was corrected for phase and baseline deformation manually using Topspin 2.1(Bruker Biospin) and the chemical shift (α-glucose at δ 5.237). The spectral region (0.4–8.6) was integrated into bins with a width of 0.002 ppm using the AMIX package (v3.9.2, Bruker Biospin). Some noise signals, such as water signals (δ 4.200–5.152) were removed. The areas of all bins were then normalized to the volume. The normalized data was used for multivariate analysis, and the model was constructed using the orthogonal projection to latent structure-discriminant analysis (O-PLS-DA) with unit variance (UV) scaling and validated with a 7-fold cross-validation method using soft independent modeling by class analogy (SIMCA)-P1(van 12.0, Umetrics, Sweden). The parameter R^2^Y is indication of the Y variables being explained by the model and Q^2^ represents the predictability of the model. The significance of the model was also validated by CV-ANOVA (*p* < 0.05). To assist the biological interpretation of the loadings generated from the model, the loadings were firstly back-transformed and then plotted with color-coded O-PLS-DA coefficients in MATLAB 7.1. The color code corresponds to the absolute value of the O-PLS-DA correlation coefficients |*r*|, which indicated the contribution of the corresponding variable to the group separation.

### Assessment of Covariates

The gender, birth date, and years of professional training of all participants were obtained with a questionnaire. Body mass index (BMI) was calculated as the weight in kilograms divided by the square of the height in meters. Body fat percentage was measured with Inbody720 (InBody Co., Ltd., Seoul, South Korea). Physical performance and function covariates were measured with standardized test methods (Yang and Shen, 2019) including grip, back strength, standing long jump (SLJ), standing vertical jump (SVJ), abdominal curl, vital capacity, sit-and-reach, acoustic reaction time, and quiet heart rate, which were closely related to the physical performance of swimmers. Health covariates were tested with an automatic biochemical instrument (Access2, Beckman Inc., United States) including hemoglobin, erythropoietin (EPO) and myoglobin (MYO).

### Statistical Analysis

Continuous variables of baseline characteristics and physical performance were presented as mean (standard deviation, SD) or median (with interquartile range, IQR), and categorical variables were expressed as frequencies (%) when appropriate. The Student’s t-test or Mann–Whitney U test was used to compare the continuous variables, and the Pearson’s ${\text{ χ}}^{2}$ test was used for comparisons of categorical variables.

The Student’s t-test was used to compare the serum metabolites between elite and sub-elite swimmers, and to determine the significantly different metabolites. Using the method of Lasso regression, the obtained significant different metabolites were screened to further be reduced. Based on the results of Lasso regression, logistic regression was performed using the R package “glmnet” for dimensionality reduction to select metabolomic markers (Friedman et al., 2010). Correlation analysis was performed between the significant metabolites and the baseline characteristics. Based on the selected metabolites and different covariates, three models were established using multivariate logistic regression, model 1 was unadjusted by any covariate, model 2 was adjusted by baseline covariates, and model 3 was adjusted by baseline and physical performance covariates. The receiver-operating characteristic (ROC) curve was analyzed for every model, with area under curve (AUC) calculated to evaluate its effect for identification and prediction of athletic status. To avoid biased estimation, average values of AUC were generated from 10-fold cross-validation (the dataset was randomly divided into ten parts, using nine of them in turn as the training set and one as the test set) in ROC analysis (Cui et al., 2020). The optimal combination of specificity and sensitivity was determined by the Youden index method (Youden, 1950).

All the above analyses were used with the IBM-SPSS 24.0 for WINDOWS and R Studio (R core 3.5.3), and the differences were considered to be statistically significant when *p* < 0.05.

## Results

### Baseline Characteristics and Covariates of Swimmers

The baseline characteristics, physical performance, and health indicators of all participants by athletic statuses were summarized in Table 1. Their average age (SD) was 19.3 (2.7) years, and 56.1% of them were men. Interestingly, sub-elite level swimmers had longer trained years than elite level swimmers (*p* < 0.001). Elite level swimmers had higher values in physical performance covariates of abdominal curl/min and sit-and-reach (*p* < 0.001), and lower values in BMI, body fat percentage, vital capacity, and resting heart rate (with *p* < 0.05, *p* < 0.01, and *p* < 0.001, respectively) than sub-elite level swimmers. Other baseline characteristics, physical performance, and health covariates were not significantly different between the two groups (*p* > 0.05).

**TABLE 1:** Baseline, physical performance and health characteristics of swimmers.

Characteristics	All subjects	Elite level swimmers	Sub-elite level swimmers	*p* value
Participants, n (%)	187 (100.0)	103 (55.1)	84 (44.9)	--
Gender				
Male, n (%)	105 (100)	53 (50.5)	52 (49.5)	0.152
Female, n (%)	82 (100)	50 (61.0)	32 (39.0)	
Age, years, mean (SD)	19.3 (2.7)	19.0 (3.3)	19.5 (1.7)	0.167
Years of professional training, median (IQR)	7.3 (4.7,11.5)	6.2 (3.9,7.7)	11.1 (7.3,13.1)	**<0.001**
BMI, kg/m^2^, mean (SD)	22.5 (2.6)	22.1 (2.2)	22.9 (2.9)	**0.027**
Body fat percentage, %, mean (SD)	16.8 (6.5)	15.4 (5.9)	18.6 (6.7)	**0.001**
Grip, kg, mean (SD)	39.1 (10.1)	39.3 (10.4)	38.9 (9.8)	0.761
Back strength, kg, mean (SD)	100.6 (28.8)	103.3 (28.9)	97.3 (28.4)	0.167
SLJ, cm, mean (SD)	224.7 (31.2)	228.6 (31.7)	220.0 (30.1)	0.064
SVJ, cm, mean (SD)	38.7 (8.4)	39.2 (8.8)	38.2 (7.9)	0.458
Abdominal curl, n/min, mean (SD)	55.2 (8.6)	57.7 (8.2)	52.1 (8.1)	**<0.001**
Vital capacity, liter, mean (SD)	5.1 (1.2)	4.8 (1.1)	5.4 (1.1)	**<0.001**
Sit-and-reach, cm, mean (SD)	20.0 (8.3)	22.0 (8.1)	17.6 (7.9)	**<0.001**
Acoustic reaction time, ms, mean (SD)	260.6 (28.1)	258.0 (26.8)	263.7 (29.5)	0.169
Resting heart rate, n/min, mean (SD)	73.1 (11.2)	71.1 (11.0)	75.8 (10.9)	**0.005**
Hemoglobin, g/l, mean (SD)	141.4 (14.7)	139.8 (14.9)	143.4 (14.3)	0.095
EPO, mIU/ml, mean (SD)	8.6 (3.4)	8.4 (2.5)	8.8 (4.2)	0.446
MYO, ng/ml, median (IQR)	19.3 (15.8,23.4)	18.9 (15.7,22.5)	19.8 (16.0,24.3)	0.141

BMI, body mass index; SLJ, standing long jump; SVJ, standing vertical jump; EPO, erythropoietin; MYO, myoglobin; SD, standard deviation; IQR, interquartile range. p < 0.05 marked in bold.

### Analysis of NMR Spectra and Significant Metabolites

Typical ^1^H NMR spectra of serum samples were obtained from elite level and sub-elite level swimmers (Supplementary Figure S1). Resonance peaks were assigned to specific metabolites based on published data and 2D NMR spectra with further confirmation by using public databases HMDB (human metabolome database) and BMRB (biological magnetic resonance bank) (Jiang et al., 2012; Song et al., 2015). 36 metabolites were assigned, involving multiple metabolic pathways such as carbohydrates, amino acids, and nucleotides (Supplementary Table S1).

**FIGURE 1:**
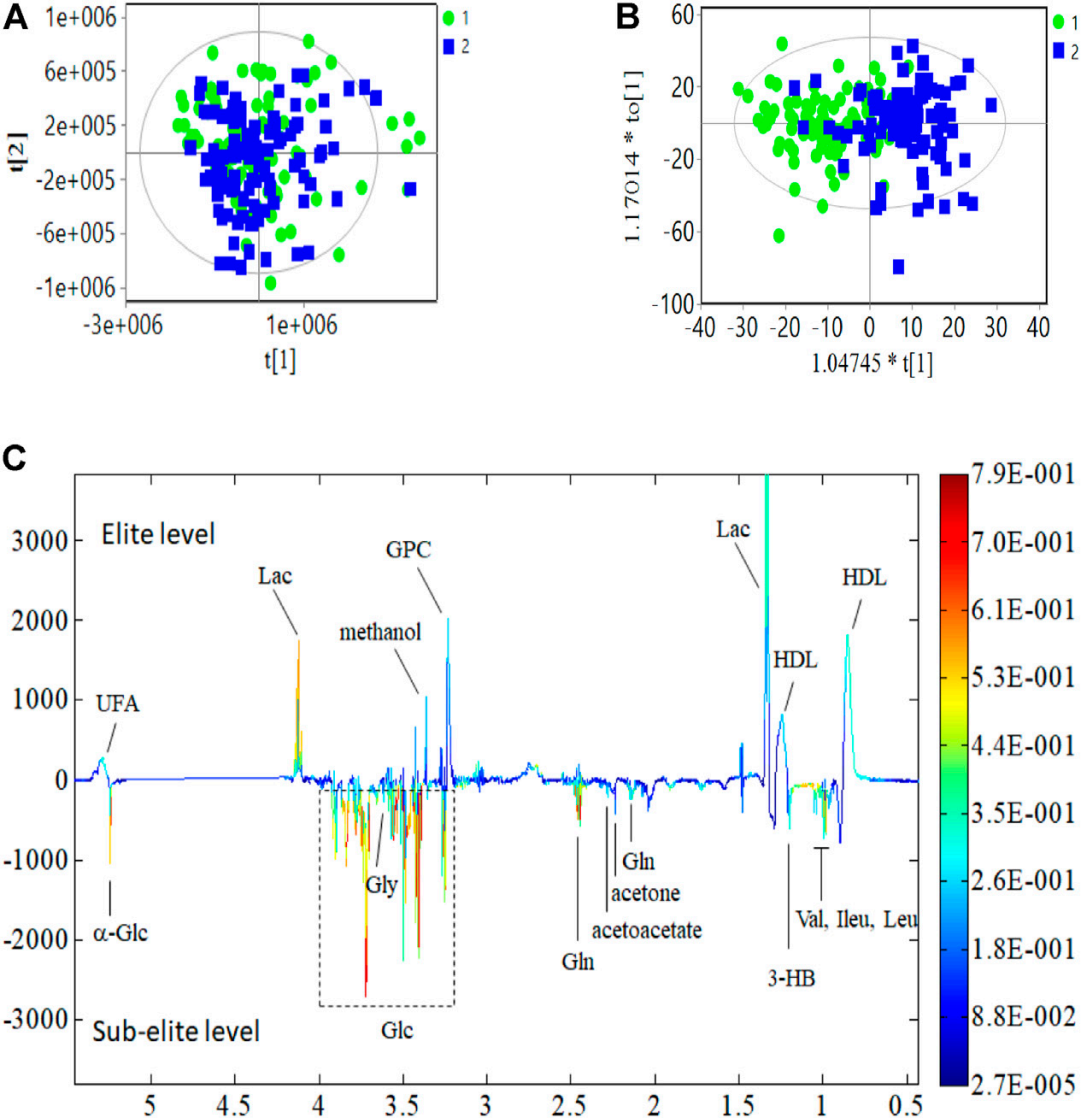
PCA, O-PLS-DA analysis and coefficient of metabolites. **(A)** PCA analysis. **(B)** O-PLS-DA score plot; R^2^ = 0.551, Q^2^ = 0.417 (*p* < 0.001); **(C)** Multicolor loading graph of multivariate metabolites analysis. 1: sub-elite level swimmers, 2: elite level swimmers

The differences in serum metabolite profiles between elite and sub-elite level swimmers were investigated using the SIMCA statistical methods of PCA (Figure 1A) and O-PLS-DA analysis (*R* ^2^ = 0.551, *Q* ^2^ = 0.417, *p* < 0.001) (Figure 1 B).The coefficient plots showed that there were 14 metabolites with distinct patterns between the two groups, including high-density lipoprotein (HDL), leucine (Leu), isoleucine (Ileu), valine (Val), 3-hydroxybutyric acid (3-HB), lactic acid (Lac), acetone, acetoacetate, glutamine (Gln), Glycerophosphorylcholine (GPC), methanol, Glycine (Gly), α-glucose (α-Glc), and unsaturated fatty acid (UFA), but lower concentrations of Glycine (Gly) and α-glucose (α-Glc) (Figure 1C). If only male athletes were included, there are 10 metabolites that showed distinct patterns, including HDL, Ileu, Val, 3-HB, Lac, Gln, methanol, Gly, a-Glc, and UFA (Supplementary Figure S2); when only female athletes were included, 11 metabolites were different between the two groups, including HDL, 3-HB, Lac, acetone, acetoacetate, Gln, GPC, methanol, Gly, a-Glc, and UFA (Supplementary Figure S3). Since the metabolites screened out by male and female gender were all included in the 14 metabolites screened regardless of gender, the subsequent analysis included all the 14 metabolites.

### Metabolites Selection and Establishment of Discrimination Models

**FIGURE 2:**
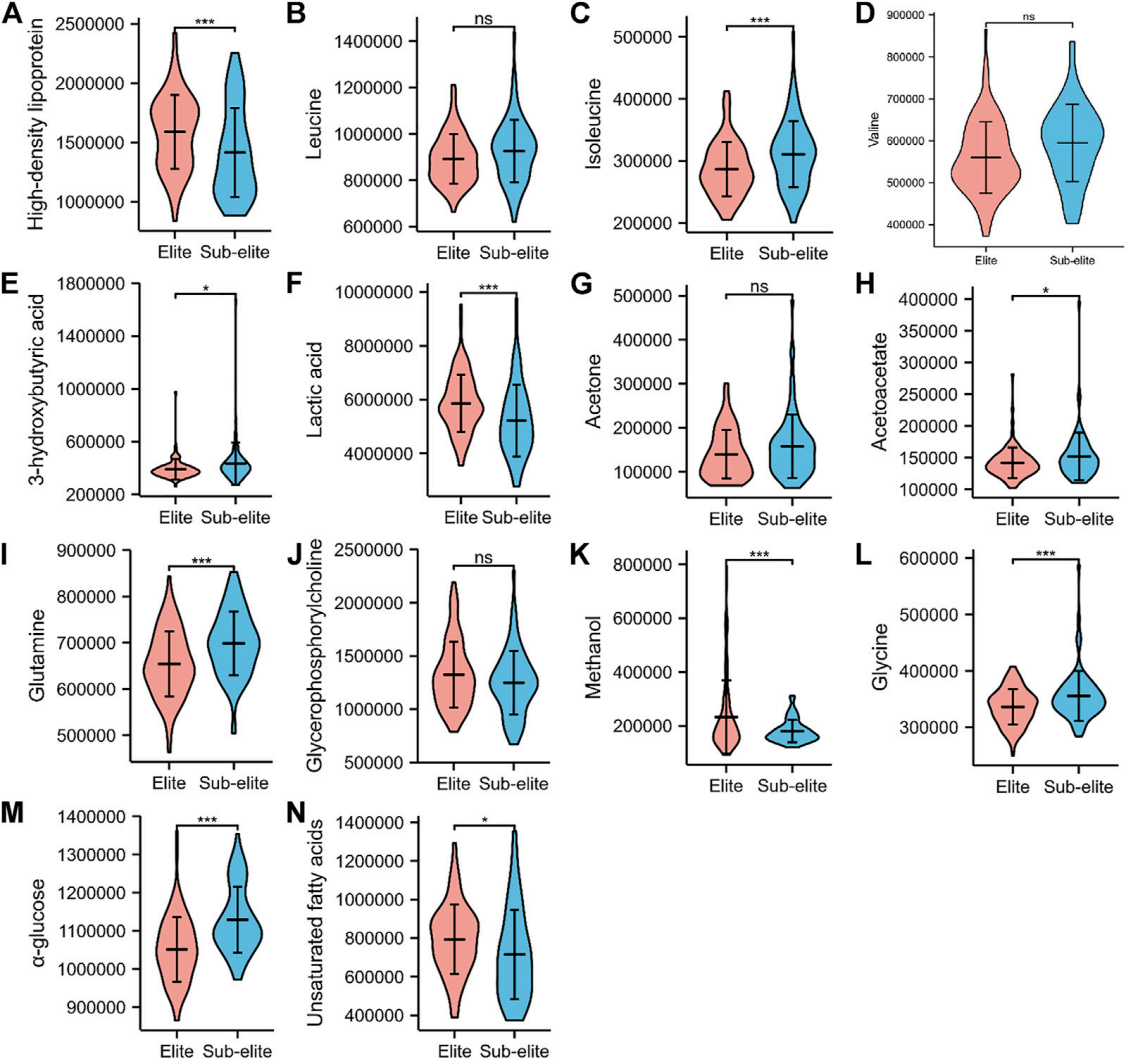
The violin plots of serum metabolites with significant differences between elite and sub-elite level swimmers. **(A)** Comparison of High-density lipoprotein between Elite and Sub-elite swimmers. **(B)** Comparison of Leucine between Elite and Sub-elite swimmers. **(C)** Comparison of Isoleucine between Elite and Sub-elite swimmers. **(D)** Comparison of Valine between Elite and Sub-elite swimmers. **(E)** Comparison of 3-Hydroxybutyric acid between Elite and Sub-elite swimmers. **(F)** Comparison of Lactic acid between Elite and Sub-elite swimmers. **(G)** Comparison of Acetone between Elite and Sub-elite swimmers. **(H)** Comparison of Acetoacetate between Elite and Sub-elite swimmers. **(I)** Comparison of Glutamine between Elite and Sub-elite swimmers. **(J)** Comparison of Glycerophosphorylcholine between Elite and Sub-elite swimmers. **(K)** Comparison of Methanol between Elite and Sub-elite swimmers. **(L)** Comparison of Glycine between Elite and Sub-elite swimmers. **(M)** Comparison of α-glucose between Elite and Sub-elite swimmers. **(N)** Comparison of Unsaturated fatty acids between Elite and Sub-elite swimmers. ***p < 0.001, **p < 0.01, *p < 0.05.

Among the 14 metabolites, ten of them were differentially expressed between the elite and sub-elite level swimmers. The elite-level swimmers showed significantly higher levels of high-density lipoprotein (HDL), lactate (Lac), methanol, and UFA, but lower concentrations of isoleucine (Ileu), 3-hydroxybutyric acid (3-HB), acetoacetate, glutamine (Gln), Glycine (Gly), and α-glucose (α-Glc) (*p* < 0.05) (Table 2;Figure 2).

**TABLE 2:** Serum metabolites with significant differences between elite and sub-elite level swimmers.

Metabolites	Elite level swimmers vs. sub-elite level swimmers		
	Fold (Elite/Sub-elite)	*p* value	adj.*p* value
High-density lipoprotein	1.123	**8.63E-04**	**1.78E-03**
Leucine	0.963	5.70E-02	6.65E-02
Isoleucine	0.923	**8.89E-04**	**1.78E-03**
Valine	0.942	8.06E-02	8.28E-02
3-Hydroxybutyric acid	0.904	**3.22E-02**	**4.51E-02**
Lactic acid	1.123	**3.39E-04**	**1.19E-03**
Acetone	0.886	5.52E-02	6.65E-02
Acetoacetate	0.934	**2.78E-02**	**4.32E-02**
Glutamine	0.936	**2.44E-05**	**1.71E-04**
Glycerophosphorylcholine	1.063	8.28E-02	8.28E-02
Methanol	1.292	**2.97E-04**	**1.19E-03**
Glycine	0.945	**5.66E-04**	**1.58E-03**
α-glucose	0.931	**4.09E-09**	**5.73E-08**
Unsaturated fatty acids	1.110	**1.19E-02**	**2.08E−02**

p < 0.05 marked in bold. adj.*p* value: *p* value after FDR (false discovery rate) correction by the Benjamini Hochberg method.

These ten significantly different metabolites by athletic statuses were then analyzed by LASSO regression to screen and select candidate metabolomic biomarkers, which would be used to identify and predict the athletic status of swimmers. After the LASSO regression analysis, eight metabolites, including HDL, lac, acetone, Gln, methanol, Gly, α-Glc, and UFA, were selected for subsequent modeling analysis (Supplementary Figure S4).

After log_2_ transformation, the eight selected metabolites were taken into a multivariate logistic regression to establish models. Out of the eight metabolites, four metabolites showed significance after the multivariate logistic regression analysis. Correlation analysis was conducted between the four significant metabolites (HDL, Gln, methan,ol and α-Glc) and baseline characteristics. The four significant metabolites were significantly correlated with a small number of baseline characteristics (*p* < 0.05), but the correlation was low (Table 3).

**TABLE 3:** Correlation analysis between four significant serum metabolites and baseline characteristics.

Characteristics	High-density lipoprotein		Glutamine		Methanol		α- glucose	
	*r*	*p-value*	*r*	*p-value*	*r*	*p-value*	*r*	*p-value*
Gender	−0.083	0.259^a^	−0.276	**<0.001** ^**a**^	−0.095	0.195^a^	−0.167	**0.023** ^**a**^
Age	−0.036	0.626^a^	0.226	**0.002** ^**a**^	−0.249	**0.001** ^**a**^	0.055	0.454^a^
Years of professional training	0.048	0.519^a^	0.183	**0.013** ^**a**^	−0.129	0.078^a^	0.116	0.116^a^
BMI	0.079	0.291^a^	0.002	0.980^b^	−0.007	0.931^c^	0.149	**0.046** ^**d**^
Body fat percentage	0.004	0.955^a^	0.051	0.504^b^	−0.170	**0.023** ^**c**^	0.152	**0.042** ^**d**^
Grip	0.042	0.570^a^	0.011	0.878^b^	0.073	0.328^c^	−0.024	0.743^d^
Back strength	<0.001	0.996^a^	−0.071	0.348^b^	0.142	0.057^c^	−0.059	0.428^d^
SLJ	0.067	0.374^a^	−0.232	**0.002** ^**b**^	0.127	0.091^c^	−0.207	**0.005** ^**d**^
SVJ	0.049	0.509^a^	−0.131	0.080^b^	0.112	0.133^c^	−0.123	0.101^d^
Abdominal curl	−0.065	0.384^a^	−0.180	**0.016** ^**b**^	0.101	0.180^c^	−0.113	0.131^d^
Vital capacity	−0.231	**0.002** ^**a**^	−0.045	0.542^b^	0.031	0.674^c^	0.024	0.746^d^
Sit-and-reach	−0.047	0.527^a^	−0.053	0.478^b^	−0.008	0.914^c^	−0.017	0.817^d^
Acoustic reaction time	−0.023	0.753^a^	0.168	**0.024** ^**b**^	0.053	0.476^c^	0.120	0.106^d^
Resting heart rate	−0.028	0.713^a^	0.085	0.259^b^	−0.066	0.381^c^	0.143	0.056^d^
Hemoglobin	−0.339	**<0.001** ^**a**^	−0.138	0.064^b^	−0.125	0.091^c^	0.002	0.977^d^
EPO	−.065	0.382^a^	−0.061	0.409^b^	0.006	0.931^c^	0.064	0.390^d^
MYO	−0.133	0.072^a^	−0.043	0.564^b^	0.047	0.527^c^	−0.117	0.113^d^

*r*: correlation coefficient.

a : correlation analysis adjusted with sports level (elite and sub-elite).

b : correlation analysis adjusted with sports level, gender, age and years of professional training.

c : correlation analysis adjusted with sports level and age; d: correlation analysis adjusted with sports level and gender. p < 0.05 marked in bold.

Three models were generated, including four metabolites unadjusted or adjusted for different covariates. Model one included four metabolites without any covariates, model two was adjusted for baseline characteristics (gender, age, years of professional training, BMI, and body fat percentage) based on model 1, and model three was further adjusted for physical performance (SLJ, abdominal curl, vital capacity,y and sit-and-reach) based on model 2. In these models, the four metabolites were all independent influencing factors on athletic statuses in swimmers (*p* < 0.05) (Table 4).

**TABLE 4:** Association analysis between significant serum metabolites and athletic status in swimmers.

Metabolites	Elite athletic status			
	OR	95%CI	*p* value	AUC
High-density lipoprotein				
Model 1	10.28	3.36–31.44	**4.40E-05**	0.649
Model 2	16.63	4.56–60.75	**2.10E-05**	0.798
Model 3	21.70	4.87–96.79	**5.48E-05**	0.849
Glutamine				
Model 1	6.77E-03	4.28E-04-1.07E-01	**3.91E-04**	0.675
Model 2	2.65E-03	1.05E-04-6.68E-02	**3.13E-04**	0.770
Model 3	1.38E-02	4.52E-04-4.21E-01	**1.40E-02**	0.823
Methanol				
Model 1	3.30	1.35–8.11	**9.12E-03**	0.577
Model 2	5.17	1.74–15.33	**3.05E-03**	0.782
Model 3	4.14	1.27–13.56	**1.87E-02**	0.834
α- glucose				
Model 1	3.19E-04	7.56E-06-1.34E-02	**2.47E-05**	0.737
Model 2	3.74E-04	5.61E-06-2.50E-02	**2.31E-04**	0.806
Model 3	4.23E-04	5.52E-06-3.25E-02	**4.53E-04**	0.849

Model 1 was unadjusted for any covariate; model 2 was adjusted for age and years of professional training; model 3 was adjusted for age, years of professional training, abdominal curl and sit-and-reach. p < 0.05 marked in bold.

**FIGURE 3:**
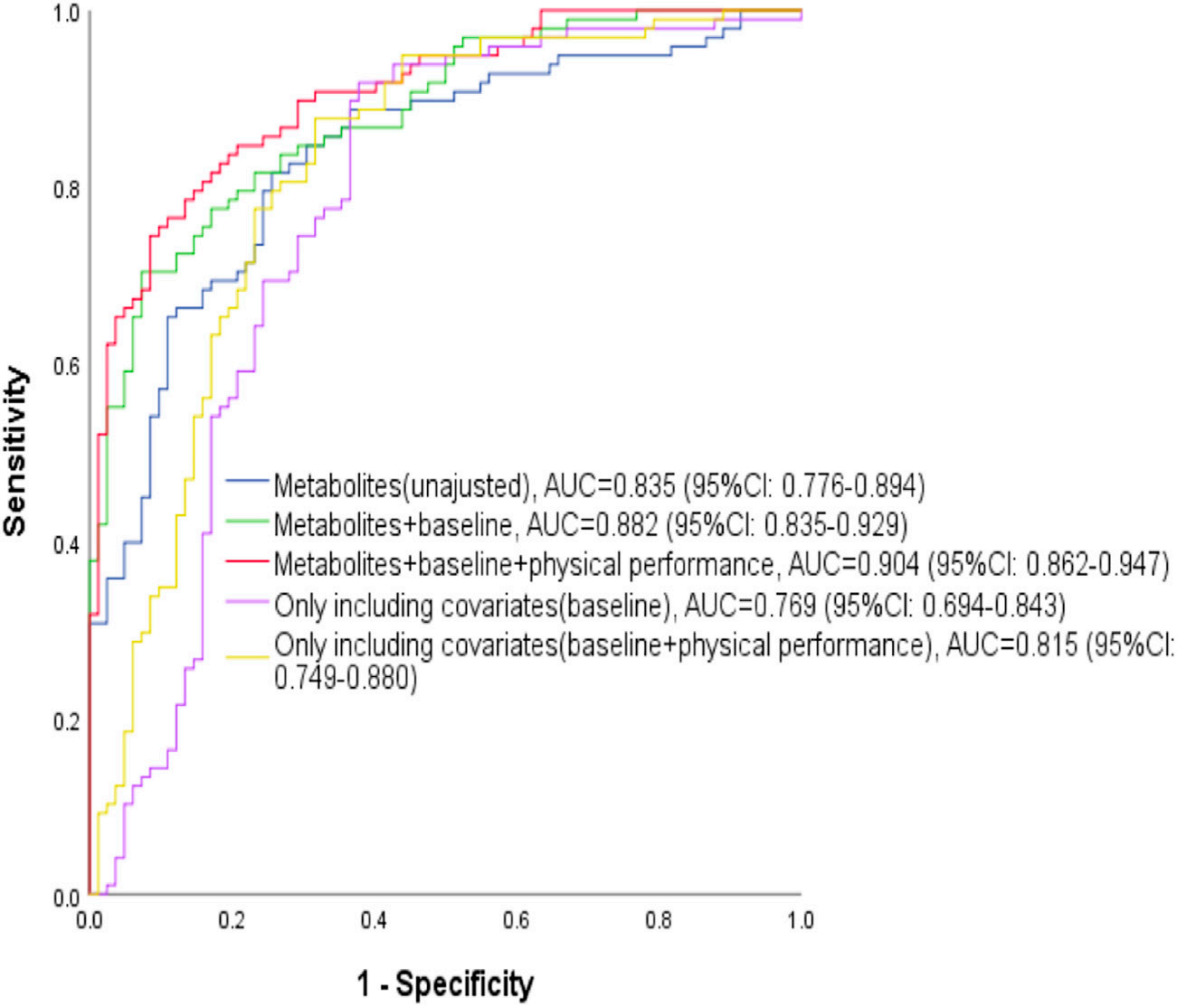
ROC analyses for identification or prediction of athletic status. The blue curve represents the ROC curve of with four metabolites of HDL, Gln, methanol and α-Glc; the green curve represents the ROC curve including the four metabolites and covariates (age and years of professional training at baseline); the red curve represents the ROC curve including the four metabolites, baseline covariates (age and years of professional training) and physical performance indicators (abdominal curl and sit-and-reach); the purple curve represents the ROC curve only including baseline covariates (age and years of professional training) without metabolites; the yellow curve represents the ROC curve only including baseline covariates (age and years of professional training) and physical performance indicators (abdominal curl and sit-and-reach) without metabolites.

The unadjusted model one identified or predicted the athletic status of swimmers reasonably well, holding an AUC of 0.835 (95% CI: 0.776-0.894) with the internal validation. The ROC curve analysis showed that the AUC increased significantly to 0.882 (95% CI: 0.835-0.929) and 0.904 (95% CI: 0.862-0. 47), when baseline characteristics (age and years of professional training) were included and baseline characteristics plus physical performance (age, years of professional training, abdominal curl and sit-and-reach) were included, respectively (Figure 3). According to the AUC value of ROC curve analysis, model three had the best identification or prediction ability in three models (AUC >0.9), of which the optimal sensitivity and specificity were 75.5 and 90.2%, respectively. Of note, when only age and year of training were included for ROC analysis, the AUC was 0.769; when all the covariates were included in ROC analysis, the AUC was 0.815. Both the AUC values were less than those with metabolites, suggesting the models with metabolites were better.

## Discussion

In this current study, using the high-throughput ^1^H-NMR method, we conducted a broad search for serum biomarkers on professional swimmers’ athletic status. We detected 36 serum metabolites with the NMR platform, most of them being amino acids. Ten of the metabolites were significantly different between elite and sub-elite swimmers, with four higher and the other six lower in the elite swimmers. After the LASSO and logistic regression analysis, four serum metabolites were identified significantly associated with the athletic status of elite swimmers. Furthermore, our study showed that the model of four metabolites adjusted for baseline characteristics and physical performance indicators could identify or predict the athletic status of swimmers reasonably well.

Metabolomics is currently widely used in many disciplines, due to its systematic, comprehensive, and high-throughput advantages (Youden, 1950; Jiang et al., 2012; Song et al., 2015; Sket et al., 2020). For example, metabolomics can be used to find trace changes in biological samples such as blood and urine, which are difficult to achieve with traditional detection and analysis techniques (Kingsbury et al., 1998). In the field of sports science, metabolomics has been demonstrated as a very promising research and analysis tool, which can not only obtain comprehensive information on athletes’ metabolites at baseline or after training (Heaney et al., 2019), but also systematically monitor the physiological state of athletes (Lavoie et al., 1983; Richard et al., 2008; Brites et al., 2017). In recent years, sports researchers have been using the methods of metabolomics to study the blood or urine metabolome characteristics of swimmers in various physiological states (Knab et al., 2013; Couto, et al., 2017; Al-Khelaifi et al., 2018; Pla et al., 2021). Among these studies, some scholars have studied the changes of the metabolic profile of elite athletes (including swimmers) in different events (Al-Khelaifi et al., 2018), some have studied the metabolic response of high-level swimmers under specific intensity training programs (Pla et al., 2021), others have studied the effects of supplementing different fresh fruit juices on chronic resting and postexercise inflammation, oxidative stress, immune function, and metabolic characteristics (Knab et al., 2013). These various studies show that it is very extensive to use metabolomics techniques and methods to study the application scenarios of swimmers. In this study, we found different levels of metabolites between swimmers of different athletic statuses, which illustrated that there were differences in the characteristics of blood metabolomics between different athletic statuses. Three out of the six metabolites lower in the elite swimmers were amino acids, including isoleucine, glutamine, and glycine. It has been previously reported that there were contrasting plasma-free amino acid patterns in elite athletes, depending on the training and fatigue status (Kingsbury et al., 1998). The higher levels of HDL and unsaturated fatty acids but lower levels of α-glucose suggested that elite swimmers probably had different substrate utilization when compared with sub-elite swimmers. An early study has reported an increase in lipid utilization in elite swimmers during a training session (Lavoie et al., 1983). Notably, both HDL and unsaturated fatty acids have been implicated with antioxidative effects (Richard et al., 2008; Brites et al., 2017).

It has been previously reported that HDL was associated with physical activity and athletic sports (Kraus et al., 2002; Valimaki et al., 2016; Fikenzer et al., 2018). It is generally believed that the HDL concentration of athletes with a certain training level is significantly higher than that of the general population. For instance, Lee H et al. (2009) found that the concentration of blood HDL of athletes with a certain training level was higher than that of the general population independent of the type of sports they were engaged in. Other researchers reported that athletes engaged in different sport disciplines showed differences in blood HDL concentration, possibly due to the different metabolic characteristics associated with aerobic and anaerobic exercise (Chou et al., 2005; Lee et al., 2009). The above reports indicated that the concentration of HDL might be related to whether or not exercise was performed, exercise style, duration, and intensity. The HDL result of our study is consistent with previous research results.

Glutamine makes up a large number of free amino acids in muscle, accounting for about 60% of the total free amino acids in the human body. Glutamine can be synthesized by glutamic acid, valine, and isoleucine. Sports practice suggested that the level of glutamine in the body could drop sharply after high-intensity strength training. If glutamine did not restore from the diet, the body will decompose muscle protein to meet its demand for glutamine. This phenomenon will not only affect the muscle volume, but also lead to the reduction of the body’s immunity (Armstrong et al., 2014). The difference in blood glutamine levels between the two athletic statuses may be related to the long-term training effects on isoleucine and valine, as we detected lower levels of those in elite swimmers as well.

Additionally, an interesting but important finding was that the level of blood methanol in elite swimmers was significantly higher than that in sub-elite swimmers. Methanol is a colorless, transparent, flammable, and volatile toxic substance. Acute methanol intoxication can damage brain function and optic nerve *via* inducing neuroinflammation (Zakharov et al., 2017; Zakharov et al., 2018). Yet methanol naturally exists in normal healthy individuals, which could be from diets, such as alcoholic beverages, fruits and vegetables; or from fermentation by gut bacteria and metabolic processes of S-adenosyl methionine (Dorokhov et al., 2015). Since the diet of the athletes has been standardized in the current study, we speculate the differential level of methanol might be likely due to the differences in metabolic methanol. However, there is still no report about the effect of the physiological concentration of methanol on sports ability. Some studies have shown that the methanol extract obtained from some special plants, such as the leaves of Eugenia species, shade dried plants, and Syzygium calophyllifolium bark, has the roles of anti-oxidant, anti-inflammatory, anti-hypertensive, anti-lipidemic, reducing blood glucose levels (Chandran et al., 2016; Aluko et al., 2019; Goldoni et al., 2019) and can even boost androgen levels (Kamran et al., 2018). Based on the aforementioned evidence, we speculate that the higher concentration of blood methanol may help swimmers improve their athletic performance. In the future, it would be interesting to explore the mechanism of higher blood content of methanol and its effects in sports capacity.

We also found the level of α-glucose was lower in elite swimmers than that in sub-elite swimmers. The α-glucose is an isomer of D-glucose, which acts as a diuretic, and detoxifier. It is generally believed that the baseline blood glucose concentration of professional athletes is lower than that of the general population (Lippi et al., 2008), which could be due to long-term high-level professional training.

In addition to the four serum metabolites finally included in the model, some other metabolites are different between the elite and the sub-elite level swimmers, such as isoleucine and valine of BCAA (branched-chain amino acids), which is also an interesting phenomenon. In our study, it was found that the concentrations of isoleucine and valine (only in male swimmers) were significantly lower in elite-level swimmers. This result was similarly reported in a recent research on elite cyclists. Cyclists with higher exercise ability were found that after a graded exercise test to exhaustion, the concentration of isoleucine in blood changed, which was significantly lower than that of athletes with lower exercise ability, but there was no significant difference in baseline test (San-Millán et al., 2020). This result may be due to the accelerated decomposition of branched-chain amino acids in blood caused by acute exercise, which are converted into Acetyl-CoA and enter the TCA cycle to participate in energy supply. The difference in exercise ability also leads to the change of metabolic profiles of branded-chain in amino acids. This was different from the results of our study. The swimmers in our study were in the basic state rather than the state after acute exercise. The reason why the baseline of cyclists had not changed may be that they were all high-level athletes, and the differences of ability were not obvious. In our study, there were still great differences in the sports ability and level between elite and sub-elite swimmers. On the contrary, the change in BCAA metabolic profiles can also cause the change in exercise training adaptability, which has been verified in animal experiments. Xu et al. (Xu et al., 2017) reported that after knocking out the gene of the enzyme that inhibits the decomposition of BCAA, mice showed higher adaptability to endurance training, and the concentration after training was also lower than that of normal mice, indicating that BCAA participated in the adaptation of endurance training. Other studies have also verified that BCAA supplementation can improve the adaptability of endurance training and have positive benefits for endurance training (Kim et al., 2013; Gervasi et al., 2020).

Another interesting phenomenon in our study was that the differences in sports level and serum metabolome also have gender characteristics. In some studies on sports ability and differential metabolites, the participants involved were generally one gender (Kim et al., 2013; San-Millán et al., 2020; Margolis et al., 2021) or a total of men and women (Gervasi et al., 2020); however, few studies focused on gender characteristics. There was a study on the changes in fat-free mass and plasma amino acids of male and female recruits after military training (Margolis et al., 2021). The study found that BCAA increased in women but did not change in men. The authors concluded that this result may be related to the differences in dietary intake, fat-free mass ratio, and energy balance between men and women. The background of this study was still different from our work, and further research can be carried out in the follow-up.

The underlying mechanisms for the different levels of metabolites among athletes at different competition levels are less known, we speculate that it could be due to differences in genetics and training regime. It is well known that genetic differences contribute to athletic capacity (Pitsiladis et al., 2016; Voisin et al., 2016; Yang et al., 2017). Of the genetic variants, *ACTN3* R577X and *ACE* I/D are two well-studied polymorphisms (Levinger et al., 2017; Yan et al., 2018). Notably, genetic variants have been reported to influence metabolic traits of elite athletes (Banting et al., 2015; Al-Khelaifi et al., 2019). More specifically, a recent genome-wide association study (GWAS) with 490 elite athletes, combined with high-resolution metabolomics profiling, reported 145 significant single nucleotide polymorphism (SNP)-metabolite associations (Al-Khelaifi et al., 2019). Moreover, four significant associations between SNPs and metabolites, were only identified in elite endurance athletes (Al-Khelaifi et al., 2019). On the other hand, the training regime is known to be different among athletes at different competition levels (Yan et al., 2016), and has been shown to influence the levels of metabolites (Yan et al., 2009).

Of note, the metabolomics results of this study were obtained *via* the NMR method, which has a lower sensitivity compared to the mass spectrometry method (Emwas et al., 2019). It would be beneficial to validate our results with the use of additional methods, such as LC-MS and GC-MS.

With the development of high-throughput detection and bio-omics technology, there are many efficient and accurate detection methods for athletes’ physical function and sports state evaluation, which enriches the original evaluation system (Gomes et al., 2020; Nieman, 2021; Sellami et al., 2022), such as evaluation of physical performance with SNPs (Yang et al., 2021a). With the popularization of detection instruments and the development of detection technology, metabolomics has greatly reduced the cost and improved accuracy. It can detect more trace metabolites and find some significantly changed trace metabolites, which are difficult to find changes by traditional methods (Heaney et al., 2019; Schranner et al., 2020). In this study, the AUC value of the evaluation model established by using the changes of trace metabolites of human serum and athletes’ physical characteristics was greatly improved, reaching more than 0.9, which has obvious advantages compared with the 0.7 level of the evaluation model with other methods (Yang et al., 2021a; 2021b). Besides, there are still some limitations in this study. Due to the scarcity of elite athletes, the sample size is relatively small, so men and women are not analyzed separately. On the validation of the model, due to the sample size, only internal cross-validation can be carried out, but not external data set validation, which is a limitation of reliability and applicability. Later, we can try to solve the problems by accumulating and sharing the samples of elite swimmers. Another limitation to this study is a static time point. In the future, it would be interesting to look at a before/after swimming exercise, or a two-week training program. We may conduct further research on the existing basis.

In conclusion, our study highlighted the potential of serum metabolomics to discover metabolite biomarkers for the athletic status of professional swimmers. Ten serum metabolites were associated with athletic status in Chinese professional swimmers. The different levels of metabolites among athletes at different competition levels could be due to differences in genetics and training regime. A four-metabolite model after being adjusted by covariates, could identify or predict swimmers’ athletic status reasonably well. Using this model with metabolite biomarkers, coaches and researchers could evaluate the competitive level at present and predict the potential of swimmers to develop to the elite level.

## Data Availability

The original contributions presented in the study are included in the article/Supplementary Materials, further inquiries can be directed to the corresponding authors.
